# HIV Infection and Compromised Mucosal Immunity: Oral Manifestations and Systemic Inflammation

**DOI:** 10.3389/fimmu.2017.00241

**Published:** 2017-03-07

**Authors:** Samantha E. Heron, Shokrollah Elahi

**Affiliations:** ^1^Faculty of Medicine and Dentistry, Department of Dentistry, University of Alberta, Edmonton, AB, Canada; ^2^Faculty of Medicine and Dentistry, Department of Medical Microbiology and Immunology, University of Alberta, Edmonton, AB, Canada

**Keywords:** HIV, mucosal immunity, oral mucosa, immune activation, periodontal disease, immunosenescence

## Abstract

Mucosal surfaces account for the vast majority of HIV transmission. In adults, HIV transmission occurs mainly by vaginal and rectal routes but rarely *via* oral route. By contrast, pediatric HIV infections could be as the result of oral route by breastfeeding. As such mucosal surfaces play a crucial role in HIV acquisition, and spread of the virus depends on its ability to cross a mucosal barrier. HIV selectively infects, depletes, and/or dysregulates multiple arms of the human immune system particularly at the mucosal sites and causes substantial irreversible damage to the mucosal barriers. This leads to microbial products translocation and subsequently hyper-immune activation. Although introduction of antiretroviral therapy (ART) has led to significant reduction in morbidity and mortality of HIV-infected patients, viral replication persists. As a result, antigen presence and immune activation are linked to “inflammaging” that attributes to a pro-inflammatory environment and the accelerated aging process in HIV patients. HIV infection is also associated with the prevalence of oral mucosal infections and dysregulation of oral microbiota, both of which may compromise the oral mucosal immunity of HIV-infected individuals. In addition, impaired oral immunity in HIV infection may predispose the patients to periodontal diseases that are associated with systemic inflammation and increased risk of cardiovascular diseases. The purpose of this review is to examine existing evidence regarding the role of innate and cellular components of the oral cavity in HIV infection and how HIV infection may drive systemic hyper-immune activation in these patients. We will also discuss current knowledge on HIV oral transmission, HIV immunosenescence in relation to the oral mucosal alterations during the course of HIV infection and periodontal disease. Finally, we discuss oral manifestations associated with HIV infection and how HIV infection and ART influence the oral microbiome. Therefore, unraveling how HIV compromises the integrity of the oral mucosal tissues and innate immune components of the oral cavity and its association with induction of chronic inflammation are critical for the development of effective preventive interventions and therapeutic strategies.

## Introduction

HIV infection continues to be a major global health issue with an estimated 36.7 million people living with HIV worldwide (1). Sadly, 1.1 million people died of HIV or HIV-related comorbidities in 2015 alone (1). The development of antiretroviral therapy (ART) in 1995 was a significant step in the fight against HIV and because of ARTs, HIV no longer is a death sentence (2). However, HIV-infected individuals successfully treated with ART do not achieve normal longevity, as the viral replication is suppressed but not completely eliminated (3–6). Long-term HIV infection, even in the presence of ART, is associated with an accelerated onset of non-HIV-related comorbidities typically related to aging, including cardiovascular diseases (CVDs), osteoporosis, cancer, neurocognitive impairment, metabolic disorders, and frailty (7–9). Viral persistence in the face of therapy has been explained by, viral latency, lowered effectiveness of drugs in some anatomical sites and cell types, and cell-to-cell spread of the virus (4). As a result, antigen presence due to HIV infection represents the major factor in the induction of inflammation and immune activation, both of which are linked to “inflammaging” (4, 10). This concept attributes to a pro-inflammatory environment and the aging process.

The persistence of immune activation and a pro-inflammatory environment is in part thought to be due to the leakiness of the gastrointestinal (GI) tract (4, 6). The GI tract is an important site of HIV pathogenesis and, early in the disease, associated with massive depletion of CD4^+^ T cells, causing irreparable destruction of the epithelial lining and damage to the immunological compartments of the GI tract (11, 12). Among CD4^+^ T-cells, T helper 17 (Th17) cells mediate immunity against pathogens at mucosal surfaces; their depletion from the gut of HIV-infected individuals increases intestinal permeability and leads to microbial translocation (13). Thus, systemic translocation of the GI tract microbial products is thought to directly stimulate the immune system and exacerbate inflammation associated with non-AIDS comorbidities (14). Microbial translocation in HIV-infected individuals has been linked to preferential depletion of lymphocytes capable of producing the effector cytokines: IL-17 and IL-22 (4, 14, 15). It is unclear as to why the gut mucosa fails to recover even after the commencement of ART. Since the gut-associated lymphoid tissue (GALT) is so similar in structure and in resident immune cells to the mucosal-associated lymphoid tissue of the oral cavity (16), it could be hypothesized that HIV infection may result in the induction of similar patterns of destruction in the oral mucosal tissues. Oral mucosal immune cells express a wide range of pathogen-recognition receptors (PRRs) and metabolic sensors that act either as suppressors or activators (17). It is unclear whether immunological changes following HIV infection compromise the integrity of physical barrier in the oral cavity and enhance microbial products translocation, which subsequently contribute in the induction of chronic inflammation. Perhaps it is possible to propose a relationship between chronic inflammation of the oral cavity, such as periodontal disease, and systemic hyper-immune activation that has been linked to accelerated aging in HIV-infected individuals.

Periodontal disease is common among adults and characterized by chronic inflammation of the oral mucosa. This disease is caused by the interplay between the pathogenic microorganisms and host defense that can lead to microbial translocation and increased risk of inflammatory conditions, such as CVD (18, 19). Studies have shown that between 50 and 70% of adults will be diagnosed with periodontal disease during their lifetime (20, 21). The prevalence of periodontal disease may result in serious oral inflammatory conditions in general, and particularly for immunocompromised individuals (22, 23). It has been shown that people living with HIV are simultaneously more likely than their HIV-negative counterparts to have serious oral health issues such as oral candidiasis, hairy leukoplakia, warts, aphthous ulcers, and herpes even in the presence of ART (24, 25). Thus, the potential impact of poor oral health in HIV-infected individuals on the induction of systemic inflammation, which could be associated with accelerated aging needs to be further studied.

Establishing the differences in the cellular and molecular components of the innate immunity of the oral cavity between HIV-infected and non-infected individuals especially in regards to activation markers, expression of PRRs, cytokines, chemokines, and host defense peptides (HDPs) may offer new opportunities to prevent oral inflammation and subsequently reduce the risk of systemic inflammation in this vulnerable population. This review focuses on the innate immune components of the oral cavity with regard to HIV infection, the key markers associated with periodontal disease, the current understanding of oral inflammation in HIV infection, as well as evaluates interventions that might be utilized to improve oral health of HIV-infected individuals.

## Innate Immune Components of Saliva

The oral cavity is a unique and sophisticated anatomical structure, characterized by a combination of soft and hard tissues, which is exposed tremendously and constitutively to external stimuli (16, 26, 27). As a result, the oral cavity is well served by numerous protective innate immune mechanisms. The oral innate immune response can be divided into secretory and cellular components. For instance, presence of numerous small and large salivary glands within the oral cavity protects us from a wide range of potential microorganisms that we are constantly exposed to through our mouth. Secretory components of the oral cavity include IgA, lysozymes, and HDPs such cathelicidins, defensins, and histatins are essential for mucosal immunity (Table 1). On the other hand, cellular innate immune components of the oral cavity are mainly epithelial cells, intraepithelial lymphocytes, granulocytes, tissue macrophages, and natural killer cells that play a crucial role in oral immunity (28). Dendritic cells (DCs) are another cellular components of the oral cavity that constantly surveying the area and are mainly found within the oral mucosa but can also be detected in the saliva (29, 30). Human saliva contains a vast amount of proteins that each has unique functions. Some proteins assist in lubrication or associate with the initiation of the digestion process, while others are involved in maintaining homeostasis and combatting pathogenic bacteria (31). Although saliva only has about 30% of the proteins normally seen in blood, it is commonly being used for diagnostic purposes in different conditions (32, 33).

**Table 1:** **Important antimicrobial peptides in the oral cavity**.

Antimicrobial peptide	Produced by	Activity	Reference
Secretory IgA	Plasma cells involved in mucosal-associated lymphoid tissue B and T lymphocytes	Major antibody in saliva Inhibits microbial adherence Agglutinates bacteria Neutralizes virus	(34–37)
Cathelicidins	Neutrophils Monocytes T cells	Antifungal, antiviral, antiparasitic Broad-spectrum activity against Gram-positive, Gram-negative bacteria and HIV	(38–43)
Defensins	Azurophilic granules of neutrophils Epithelial cells	Antimicrobial against Gram-positive and Gram-negative bacteria, HIV, mycobacteria, and fungi Induce TLR signaling and recruitment/activation of dendritic cells	(17, 38, 40, 44, 45)
Histatins	Component of saliva synthesized by parotid and submandibular salivary duct cells	Potent activity against fungi (including *Candida albicans*) Regulate oral hemostasis Bond metal ions in saliva	(38, 43, 46)
Lactoferrin	Exocrine glands Neutrophils in infected/inflamed sites	Binds iron Bacteriostatic Bacteriocidal, anti-HIV Decreases biofilm formation Decreases reactive oxygen formation	(35, 40, 47–51)
Lysozyme	Present in saliva Cytoplasmic granules of macrophages and polymorphonuclear neutrophils	Antibacterial Antiviral, anti-HIV Binds to and aggregates Gram-positive bacteria	(35, 40, 48, 51)
Secretory leukocyte protease inhibitor	Component of saliva Produced by neutrophils, macrophages, submandibular glands	Antiviral, anti-HIV Inhibits proteases Inhibition of neutrophil elastase Bactericidal, antifungal	(40, 52–54)

Whole saliva is made up oral fluids from the major and minor salivary glands as well as from the gingival crevicular fluid and serum transudate from the mucosa (55). It may also contain fluids from areas of inflammation, immune and epithelial cells, food debris, and microbes (55, 56).

Saliva can be collected with or without stimulation. Saliva that is stimulated can have a different pH value when compared to unstimulated saliva, as well as has an increased volume of water content (55). However, unstimulated saliva can be difficult to collect if the individual does not produce a lot of saliva, either from dehydration, diet, age, gender, or from the side effects of medications (56). In 2010, Miller et al. found that unstimulated whole saliva allowed for the identification of several different biomarkers that were relevant to the assessment of cardiovascular and periodontal disease (55) (Table 2). These biomarkers are generally detected at lower levels than found in serum; however, there are some that appear at higher concentrations. Common detected analytes include tumor necrosis factor (TNF)-α, IL-1β, IL-6, fractalkine, growth-regulated oncogene 1-α, and monocyte chemotactic protein-1 (55). Elevation of IL-6, osteonectin, IL-1β, IL-8, MIP-1α, and MIP-1β in the saliva in acute inflammation has been reported and are elevated in the saliva of subjects 6–9 months prior to detection of bone loss compared to the saliva of individuals who remained healthy (57, 58).

**Table 2:** **Abundant analytes present in oral cavity**.

Analyte	Activity	Levels in saliva	Reference
Tumor necrosis factor (TNF)-α	Remodels tissue Recruits inflammatory cells Initiates bones resorption and inhibits bone collagen synthesis	Increased in patients with chronic periodontitis	(55, 59–63)
IL-1β	Induces activation of osteoclasts; resorbs bone Synthesized and secreted by fibroblasts, endothelial cells, and infiltrating leukocytes	Elevated levels in patients with increased bone loss However, some patients with no evidence of bone loss presented with high levels	(55, 57, 58, 64–66)
IL-6	Produced in infectious or stressed environment T and B cell growth Activation of osteoclasts	Increased expression in alveolar bone loss patients	(55, 57, 58, 65, 66)
Fractalkine (CX_3_CL1)	Induces adhesion and migration of leukocytes Chemotactic activity for T cells and monocytes	Increased in periodontal disease Upregulated by pathogen-associated molecular patterns	(67–72)
CXCL1	Involved in both inflammation/proliferation Attracts neutrophils and induces their degranulation	May be involved in recruiting lymphocytes during diseased state of periodontitis	(72, 73)
Monocyte chemotactic protein-1/CCL-2	Induces chemotaxis of monocytes Increases calcium influx Stimulates expression of integrins Produced by endothelial cells	Found to increase in patients with periodontal disease, mostly in the GCF but can be detected in the saliva as well	(63, 74–79)
Osteonectin	Produced by osteoblasts Binds calcium in bone in addition to strongly binding collagen and hydroxyapatite	Lower levels in patients with periodontal disease and higher levels in patients with healthy bone levels	(57, 80)
IL-8/CXCL-8	Attracts and activates neutrophils	Increased in patients with chronic inflammation Higher than IL-6 and TNF-α	(66, 81)
MIP-1α/1β/CCL-3	Secreted by inflammatory cells Related to cell adhesion and migration Stimulates monocytes and osteoclast progenitor cells to absorb bone	Elevated levels in saliva in patients susceptible to bone loss Elevated in GCF of specific teeth can indicate bone loss	(55, 58, 63, 82, 83)

There is some concern as to whether salivary proteases may degrade cytokines and decrease their ability to be detected; however, several studies have shown that the addition of protease inhibitors prior to freezing of saliva specimens does not impact the levels of detectable biomarkers in the saliva (56, 57). In addition, it has been reported that these inhibitors may interfere with the downstream analytical biomarker assays (84). It has also been shown that protein degradation in saliva is a rapid process, and the protein breakdown was not affected by the addition of sodium azide to the saliva samples (85). Similar observations were made following incubation with or without protease inhibitor. For instance, the addition of a protease inhibitor reduced detectable biomarkers from 26 to 19 compared with saliva samples when no inhibitor was added (85). This determines that protein degradation still occurs even in the presence of inhibitors, and therefore, saliva centrifugation and freezing the supernatant at −80°C as soon as possible is the best approach for maintaining its integrity (56, 84–86).

In addition to their main role in immunoregulation, these cytokines and proteins that are produced by infiltrating immune cells stimulate local osteoclasts which, in turn, induce alveolar bone resorption. Chronic inflammation of the alveolar mucosa is initiated by the intricate subgingival biofilm made up of opportunistic microorganisms and commensal bacteria (87). In response to these pathogens, inflammatory cells and gingival resident cells release destructive cytokines and proteinases that can damage gingival ligaments and initiate bone resorption (30, 34, 47). For example, IL-1β and IL-6 are found to be directly proportional to bone loss, where osteonectin is inversely proportional to bone loss (57). IL-8 is found at higher amounts than IL-6 and IL-1β in bone loss patients; however, MIP-1α may be the best biomarker to identify periodontal disease (58). In the elderly population, these markers will indicate the presence of periodontitis, since periodontal disease is more common in aging patients (88–90).

## Innate Immune Components of the Oral Cavity and HIV

HIV infection appears to directly or indirectly impact the systemic and local innate immunity leading to oral opportunistic infections and malignancies (91). For instance, higher expression of oral cytokeratin in HIV-infected individuals and patients on ART has been reported, which indicates transformation of the oral mucosa and increased risk of malignancy (92).

**Figure 1:**
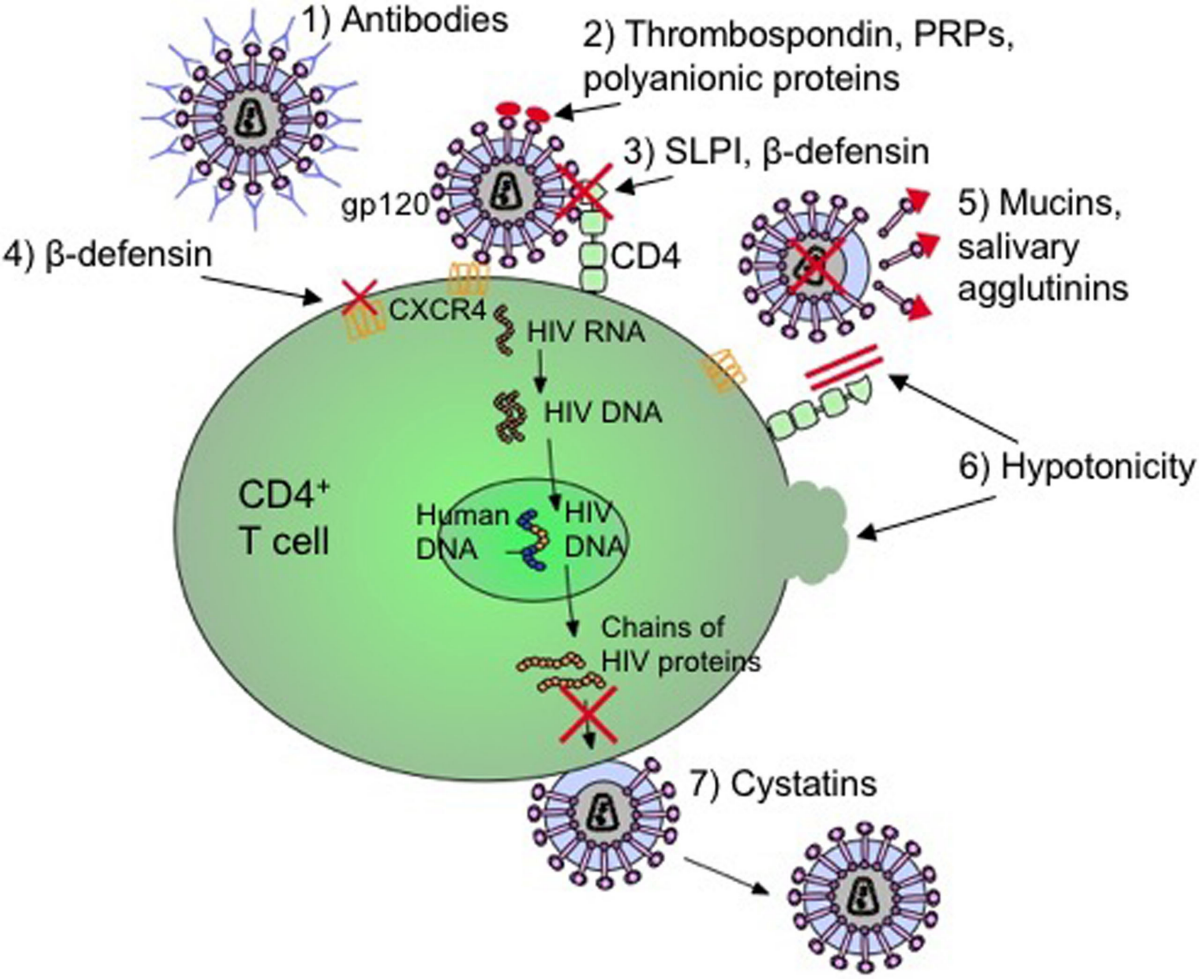
**Mechanisms by which salivary inhibitors may disrupt HIV-1 infection**. (1) Virus-specific antibodies (predominantly IgA) neutralize free-floating virus. (2) Thrombospondin, acidic proline-rich proteins (PRPs), and polyanionic proteins block cell binding by interfering with gp120 on the virus. (3) Secretory leukocyte protease inhibitor (SLPI) and β-defensin disrupt the virion from binding to T cell or macrophage. (4) β-defensin also internalizes and downregulates CXCR4 coreceptor to deny cellular entry. (5) Mucins and salivary agglutinins bind to gp120 and remove it from the virion making it defective. (6) The hypotonicity of the saliva lyses infected cells as well as physically blocking the CD4 coreceptor. (7) Cystatins interfere with proteolytic processes by inhibiting viral cysteine proteases (17, 34, 44, 101–110).

As indicated in Figure 1, there are several secretory components of saliva that has antiviral and specifically anti-HIV activities. These components include cathelicidins, defensins, lactoferrin, lysozyme, and secretory leukocyte protease inhibitor (SLPI) (38–41, 44, 45, 48, 49, 52–54, 93–95). Cathelicidins are small cationic peptides that express antimicrobial activity and found in several tissues including the lungs, intestine, mucosa, skin, and oral cavity (41). They are predominantly found in the granules of neutrophils, mast cells, lymphocytes, keratinocytes, and epithelial cells (41). A domain of cathelicidin, LL-37, inhibits HIV replication in peripheral blood mononuclear cells and is currently being evaluated to determine if the same domain inhibits HIV in saliva (41, 96). Human β-defensins (hBD) are constitutively expressed by oral epithelium and some immune cells and hBD-2 and -3 are shown to inhibit HIV replication (45). Defensins inhibit HIV by directly binding to the virus as well as by downregulating the expression of CXCR4 on the cell surface (45). Lactoferrin is an iron-binding glycoprotein with strong anti-HIV activity (97). In one study, Kazmi et al. incubated whole saliva with an antilactoferrin antibody and determined that there was a reduction in HIV inhibition activity by 65% when compared to incubation with an IgG control (97). However, the exact mechanism by which lactoferrin inhibits HIV in saliva is still unclear. Lysozyme is another components of the saliva with antiviral activity. It is produced by the salivary glands, in particular the sublingual salivary gland, as well as by neutrophils, macrophages, and is found in the gingival crevicular fluids (98). It is speculated that the antiviral activity of lysozyme may be due to the degradation of viral polysaccharides and RNA transcripts (99). SLPI is a ubiquitous protein produced by macrophages, neutrophils, epithelial cells, and the acinar cells of parotid, submandibular, and submucosal glands (40). It is ambiguous as to how SLPI inhibits HIV infection in the saliva; however, it appears to involve the host cell instead of binding to the virus (53). SLPI may inhibit a step of viral infection that occurs once the virus has bound to the cell but before reverse transcription occurs (100).

Salivary function and composition are altered with HIV infection and some salivary components such as lactoferrin and IgA levels are altered in HIV patients with oral candidiasis compared with healthy subjects (111, 112). For example, frequent oral infections in HIV patients have been linked to significant reduction in lactoferrin and secretory IgA production by parotid glands (113).

Thus, innate immune salivary components exhibit a wide range of anti-HIV activities; however, any deficiency in their secretion and activity may enhance HIV-replication/acquisition in the oral cavity.

## Immuno-Phenotypes of the Oral Cavity

The oral epithelium provides a physical barrier that protects underlying tissues from infection by pathogenic microorganisms as well as the effects of environmental threats. The oral mucosa is the port of entry into the GI tract where immune homeostasis is an important feature of the epithelial lining. The high permeability of the oral mucosa results in consistent contact between potentially harmful and harmless antigens with resident immune cells (27). As we mentioned earlier, the innate immune cells such as macrophages, natural killer cells, polymorphonuclear leukocytes, and DCs are present in the oral cavity and play important roles in mucosal immune tolerance (26).

Dendritic cells are professional antigen-presenting cells (APCs) that are divided into two subpopulations: myeloid DCs and plasmacytoid DCs (26, 114) (Table 3). These two populations of DCs differ in their expression of PRRs, in the types of cytokines they produce, and their mechanism of activating T cells. Myeloid DCs can be further divided into CD1c^+^ DCs and CD141^+^ DCs (26). A subset of CD11c^+^ myeloid cells are CD14^+^, or interstitial DCs (114). These DCs are more like monocytes or macrophages than conventional CD1c^+^ or CD141^+^ myeloid DCs and can express CD14^+^ but do not express costimulatory molecules (114). Langerhans cells (LCs), a subset of myeloid DCs, are positive for CD11c, MHC-II, as well as CD207 (langerin) (27). Oral LCs are positive for all the LC markers as well as CD1a. Oral LCs are permanent residents of the basal layer of the oral epithelium and are crucial in maintaining immune tolerance (115). Oral DCs can also express costimulatory molecules CD80 (B7.1), CD86 (B7.2), and CD40 as markers of activation (27). Plasmacytoid DCs generally lack all of the myeloid antigens (114). Identification of plasmacytoid DCs in healthy oral tissues has been a challenge, but they can be detected in inflammatory conditions in the oral cavity (116).

**Table 3:** **Classification of dendritic cells (DCs)**.

DCs	Markers expressed	Negative markers	Reference
Myeloid CD1c^+^ (conventional) DCs	CD1c, CD11b, CD11c, CD13, CD33, HLA-DR	CD14, CD16, CD19, CD20, CD68	(114, 117, 118)
Myeloid CD141^+^ (conventional) DCs	CD141, CD11c, CD13, CD33, HLA-DR	CD11b, CD14, CD68	(114, 117, 118)
Interstitial DCs	CD11c, CD14, CD163, CD209, HLA-DR	CD1c, CD141, costimulatory molecules	(114)
Langerhans cells	CD1a, CD11b, CD11c, CD32, CD45, CD80, CD86, CD207 (langerin), HLA-DR	CD14, CD141, CD163, CD205, CD209	(29, 114, 118, 119)
Plasmacytoid DCs	CD2, CD7, CD45RA, CD68, CD123, CD303, CD304, HLA-DR	CD11b, CD11c, CD13, CD14, CD33	(114, 117)

Mucosal DCs are the important component of oral tolerance as they have to maintain a homeostatic environment. DCs have to initiate a proper immune response against foreign and invasive pathogens, while preventing unnecessary activation of the immune system in response to commensal bacteria or food particles. Resident DCs are found throughout the oral mucosal epithelium but are most abundant in the vestibule, buccal mucosa, and hard palate (27). The morphology and density of DCs are proportional to age and tend to decrease as age increases; however, the number of DCs and maturation stage increases in the presence of inflammation (120).

While the oral cavity is enriched with APCs due to the abundance of food and microbial antigens, T cells are seen in very low frequency in the saliva and at the oral mucosal surfaces but B cells are almost absent in healthy patients. However, in the buccal tissues, CD4^+^ helper T cells are the dominant cell populations followed by CD8^+^ T cells and a small percentage of γδ T cells (121). We have already shown that γδ T cells are abundant in the oral cavity and play a protective role against oral candidiasis, which is one of the opportunistic fungal diseases associated with HIV infection (122). Among these CD4^+^ T cells, about 10–15% of them are Foxp3^+^, assumed to be T regulatory cells (Tregs) (121). This is important to mention that Foxp3 expression does not represent Tregs in human since activated CD4^+^ T cells may also express this marker. High levels of IFN-γ were found to be produced by both oral CD4^+^ and CD8^+^ T cells, but only 1–2% of CD4^+^ T cells secrete IL-17 (121). However, IL-13 and IL-22 were not detected in healthy oral mucosa (121). When comparing healthy controls to individuals with periodontal disease, it was found that CD3^+^ T cells remained the largest population; and interestingly the B cell population, which was nearly absent in the healthy population, became evident in the individuals with periodontal disease (121).

Neutrophils play a crucial role in innate defense as they contain and eliminate invading organisms after breaching host anatomical barriers, such as the skin and mucosa. Although they are found in abundance in the oral cavity of healthy individuals, periodontal disease results in a significant increase in CD15^+^CD16^+^ neutrophils in the oral cavity (121). They are required to ensure homeostatic conditions in the oral cavity. A harmonic balance of neutrophils is crucial in order to keep bacterial numbers in check as too many or too few neutrophils can lead to periodontal tissue damage (123). An increase in neutrophils is linked to an upregulation of IL-17, which has been shown to be associated with bone loss in animal models of periodontal disease (124). Although secretion of IL-17 is predominantly produced by CD4^+^ T cells (121), secretion of IL-17 by CD4^+^ T cells creates a positive feedback loop in the area of inflammation as IL-17 promotes neutrophil recruitment and also attracts more IL-17-producing CD4^+^ T cells (123). This generates a vicious cycle where inflammation persists indefinitely. In addition to the secretion of IL-17 by CD4^+^ T cells, they also produce an array of Th1- and Th2-type cytokines such as IFN-γ, IL-6, IL-13, and sometimes IL-10 (125–127). IL-6 and IFN-γ are pro-inflammatory cytokines that have been linked to the stimulation of osteoclastic resorption in periodontitis (126–128). IL-10 and IL-13 are both anti-inflammatory cytokines, which serve to inhibit bone resorption (101). This balance of cytokines ensures homeostasis of the periodontium; however, when this balance is disrupted by pathogens such as *Porphyromonas gingivalis* or *Aggregatibacter actinomycetemcomitans*, the paradigm can be shifted toward a Th1 response and induction of bone resorption (126, 128). This indicates that an increase in activated CD4^+^ T cells is associated with bone loss and disease progression.

The innate immune cells are the first line of defense against invasive pathogens and rely on a large group of PRRs, which discriminate different pathogens through recognition of conserved evolutionary molecular motifs, called pathogen-associated molecular patterns (PAMPs) (101). Among the PRRs, TLRs have been studied most widely. Upon PAMP engagement, PRRs trigger intracellular signaling cascades eventually culminating in the expression of a wide range of pro-inflammatory molecules, which together orchestrate the innate immune response to infection, and also is a prerequisite for the subsequent induction of adaptive immunity (101, 129, 130). TLRs are expressed by various cells such as endothelial cells, epithelial cells, gingival fibroblasts, and several APCs (101).

Of the 10 TLRs that are known to exist in humans, TLR2 and TLR4 are seen most abundantly in the oral cavity (130–133). TLR4 recognizes LPS, where TLR2, along with TLR1/TLR6 recognizes lipopeptides, peptidoglycans, lipotechoic acid, and unmodified protein structures (130, 134, 135). Evidence suggests that both TLR2- and TLR4-positive cells are upregulated in the gingival tissues in periodontitis (17, 130, 131, 133). Binding of TLR2 and TLR4 to PAMPs presented on pathogens will induce the expression of ICAM-1 and LFA-1 in the oral cavity which can lead to downstream bone resorption and increase in the rate of periodontitis (101).

Nucleotide-binding oligomerization domain-like receptors (NOD-like receptors) are another member of PRRs. They are intracellular sensors that detect PAMPs and endogenous molecules and can be upregulated similar to TLRs upon stimulation by bacterial components (102, 136). They can also sense danger-associated molecular pattern molecules that are associated with cell stress and can induce an inflammatory response in the absence of infection (102). NOD1 is ubiquitously expressed within various cells such as mononuclear cells, macrophages, DCs, and intestinal epithelial cells, while NOD2 is expressed mostly in phagocytic cells and intestinal Paneth cells (102, 103). It has been shown that NOD1 is involved in the recognition of Gram-negative bacteria confirming that this receptor is expressed on epithelial cells since these types of bacteria are commonly found to threaten epithelial cell linings (104).

Oral epithelial cells constitutively express NOD1 and NOD2, which induce the production of antimicrobial products such as beta-defensin and peptidoglycan-recognition proteins (105). Interestingly, NOD1 and NOD2 do not induce pro-inflammatory cytokines when stimulated (106). This may suggest that these pattern-recognition molecules on oral epithelial cells actively contribute in the removal of bacterial components without initiating an inflammatory response in the oral mucosa (105).

The innate immune cells of the oral cavity are a crucial component in protecting underlying mucosal tissues as well as the entrance of the GI tract. During homeostasis, these cells work together seamlessly to protect the oral mucosa without initiating unnecessary inflammatory responses. Disruption of this homeostatic state by pathogenic bacteria could result in periodontitis and subsequently to the translocation of bacterial products into the bloodstream that may lead to systemic inflammation and potentially CVDs (107, 108). Therefore, any inflammatory condition in the oral cavity (e.g., periodontal disease) would result in the recruitment of different immune cells including HIV-target cells into the mucosal sites which may facilitate oral HIV-transmission. On the other hand, immune deficiency associated with HIV infection could impair innate immune responses in the oral cavity and predisposes the host to opportunistic infections.

## Oral Transmission of HIV

HIV transmission *via* the oral cavity has been a debatable concept. Multiple studies have failed to isolate the virus in the oral epithelial cells, and therefore, absence of virus in the oral cavity makes its transmission questionable (24, 28, 91, 137). Therefore, it has been thought that the risk of contracting HIV during oral sex is very low and antimicrobial peptides, such as human beta defensins and SLPI, have been implicated with the low rate of oral HIV transmission (138, 139). Orogenital transmission predominantly occurs *via* receptive oral sex; however, it seems to be very rare (95). The risk of acquiring HIV from receptive oral sex with a HIV-positive partner has been estimated to be between 0.04 and 0.06% (95). Acquiring HIV during receptive anal sex has been estimated to be 1.4% and contracting HIV *via* intravenous drug injection has been speculated at 0.63–2.4% (140, 141).

However, the controversy of oral transmission of HIV *via* receptive oral sex is continually examined. Contracting HIV *via* oral sex is difficult to determine because of lack of previous sexual history and that oral exposure rarely happens independently of other mucosal exposures. Studies investigating oral transmission of HIV have had small cohorts of high-risk individuals, making them insufficient to detect transmission by lower risk sexual behaviors (142). In one study, a homosexual couple reported engaging in only unprotected oral intercourse, and no other form of coition (143). The HIV-negative partner became seroconverted through this method; however, it is worth noting that this couple had only been sexually active for 1 month and it is unclear as to whether the seronegative partner could have unknowingly received HIV from a previous partner. Conversely, a study from 1990 to 2000 followed a cohort of heterosexual HIV-serodiscordant couples to determine if seroconversion occurred from unprotected oral intercourse (144). The 135 of 292 participants who were included in the study used condoms for vaginal or anal intercourse but did not use protection for orogenital contact. In addition, the participants confirmed that no condoms broke or slipped during penetrative intercourse. There was over 19,000 unprotected orogenital contacts in the 10-year period, looking at both receiving and performing oral sex with an infected partner, without a single case of HIV transmission occurring (144).

It has been well documented that disrupted, damaged, or inflamed oral mucosal tissues impact the risk factor for oral HIV transmission (145–147). In fact, there is a higher prevalence of contracting HIV through oral intercourse if the partner performing the act on an HIV-infected individual is a crack-cocaine user (146). Oral sores, blisters, and cuts are likely to occur from the hot smoke, hot glass or metal pipestem, or from sharp edges of the glass pipestem (146).

Although possible to contract HIV through the oral cavity, it appears that the oral innate immunity plays an important role in preventing the viral infection. This may be due to the protective role of the oral mucosa and/or the anti-HIV molecules present in saliva. For instance, SLPI is a protein found in mucosal secretions originating from submucosal glands and epithelial cells lining mucosal surfaces (137). SLPI has been found to have anti-HIV properties and disrupt the virus from infecting macrophages (53, 54). Furthermore, removal of SLPI in whole saliva *in vitro* was found to decrease its antiviral activity (53). Compared to other mucosal secretions in the body including breast milk and seminal fluid, saliva has the highest levels of SLPI, where infectious viral levels could not be detected (137).

However, HIV infection impacts expression and secretion of oral innate immune molecules. For instance, levels of SLPI in HIV-infected individuals are found to be decreased (94). In addition to SLPI, other oral secretory components including beta-defensin, salivary agglutinin, and mucins, thrombospondin, acidic proline-rich proteins (PRPs), and polyanionic proteins exhibit antimicrobial activities (52, 95). Beta-defensin blocks HIV replication by directly interacting and blocking virion activity as well as by exerting a chemokine-like effect on CD184 (CXCR4) causing this coreceptor to internalize and downregulate (45). CD184 is one of the coreceptors utilized by the virus for cellular entry of CD4^+^ T cells. Both high- and low-molecular weight mucins (MG1, MG2), as well as salivary agglutinins bind to and dislodge the viral envelope glycoprotein, gp120, from virions which form defective viral particles that cannot infect host immune cells (93, 95). Thrombospondin, PRPs, and polyanionic proteins (such as albumins) block cell binding by interfering with the interaction between the cell and gp120 (95). Other factors of preventing HIV transmission may include physical blocking of the virus due to the high viscosity or viral lysis from low hypotonicity of saliva (148). Even though these antimicrobial factors are important in preventing HIV infection in the oral cavity, SLPI is considered to be the main factor in saliva to protect against HIV infection (53, 137) (Figure 1).

Although adults rarely seem to be infected with HIV *via* the oral cavity, it does appear to be a gateway for infectious HIV in postnatal vertical transmission (24). Newborns who are not infected *in utero* could acquire HIV *via* the oral cavity by ingesting infected vaginal secretions or amniotic fluids during delivery (149). In addition, transferring HIV from mother-to-infant *via* breast milk has a risk of about 16% (150). Even if viral RNA levels are relatively small, the volume of breast milk consumed in a day is quite large and there is repetitive exposure of the oral mucosa to the virus and any micro abrasion could facilitate the HIV transmission. It is still unclear as to where the transmission occurs but studies in neonatal rhesus macaque demonstrated that the oral mucosa, tonsils, upper GI tract, and stomach as possible targets for simian immunodeficiency virus (SIV) transmission (24).

## Oral Mucosal Infections and HIV Infection

**Figure 2:**
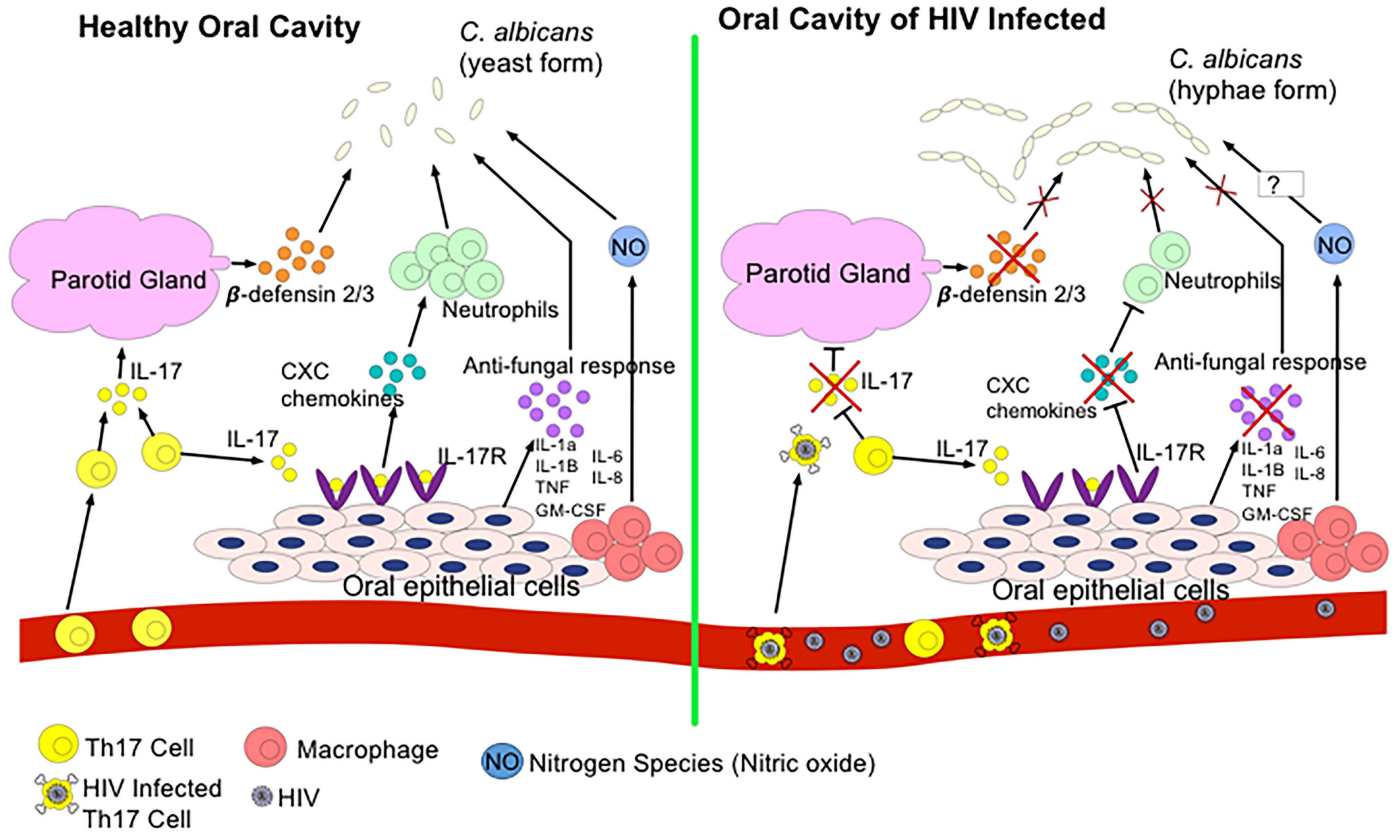
**Regulation of *Candida albicans* growth in a healthy oral cavity versus HIV-infected oral cavity**. In a state of health, T helper 17 (Th17) cells secrete IL-17 to induce the parotid gland to secrete β-defensin 2/3 in the saliva. β-defensins inhibit *C. albicans* growth. IL-17 also binds to IL-17R on oral epithelial cells to express CXC chemokines to recruit neutrophils to the oral cavity to also combat *C. albicans*. Oral epithelial cells, neutrophils, and resident macrophages secrete antifungal cytokines and nitrogen species such as nitric oxide (NO) to kill *C. albicans*. During the HIV infection, HIV infects and eliminates Th17 cells. Thus, reduction in IL-17 secretion prevents induction of β-defensins, downregulates CXC chemokines expression, and decreases production of other antifungal cytokines. As a result, transition of *C. albicans* to pathogenic hyphae form occurs.

Oral mucosal infections, such as oropharyngeal candidiasis (OPC) or hairy leukoplakia, are commonly seen in HIV-infected individuals suggesting compromised oral mucosal immunity due to HIV infection (25, 91). In fact, studies have shown that 70–90% of individuals infected with HIV will develop at least one oral manifestation over their lifetime, and that the appearance of severe oral mucosal infections can be an indicative of progression to AIDS (151, 152). The commencement of ART does not alleviate all of these manifestations. For instance, a sixfold increase in the rate of human papillomavirus (HPV) infection, more commonly known as oral warts, has been reported with the onset of ART (153, 154). It is still unclear as to why ART decreases some oral lesions but increases others. As we discussed above, Th17 cells mediate immunity against pathogens at mucosal surfaces and are key players of barrier integrity (155). Th17 cells depleted from the gut of HIV-infected individuals can lead to microbial translocation and non-AIDS comorbidities. Th17 cells along with IL-22 stimulate epithelial cells to produce antimicrobial factors to eliminate fungi such as *Candida albicans* and other bacteria by promoting inflammation through induction of inflammatory cytokines, chemokines, and recruitment of neutrophils (156). Recently, it has been reported that Th17-associated cytokines, IL-17 and IL-23, are essential in protection against OPC and IL-17-induced neutrophil recruitment plays an instrumental role in defense against mucosal candidiasis (157) (Figure 2). Therefore, in addition to immunodeficiency due to CD4^+^ T cell depletion, it is possible to speculate that possible elimination or impairment of IL-17-producing cells in the oral mucosa in the course of HIV infection might be associated with susceptibility to OPC and other opportunistic infections. In addition, production of reactive oxygen species and nitrogen radicals such as nitric oxide (NO) can damage or inhibit the growth of *C. albicans* and in agreement we have shown that NO mediates protection against oral infection with *C. albicans* (122). In the oral cavity, NO is produced by endothelia, epithelia, and macrophages as well as certain bacteria and by abiotic acidification of nitrite (158). The release of NO by these cells limits the proliferation of *C. albicans*; however, in some cases, *C. albicans* expresses flavohemoglobin genes to resist against NO (158). Since NO plays an important role against *C. albicans* in the oral cavity, studying the potential effect of HIV infection on NO production and possible mutation of flavohemoglobin genes to resist NO in HIV-infected individuals merits further investigations.

As discussed above, the most common oral manifestations associated with HIV infection are OPC and oral warts. In a study of 142 ART naïve and HIV-infected patients, oral candidiasis was found to be the most prevalent oral manifestation, followed by melanotic hyperpigmentation, and then oral hairy leukoplakia (159). Once ART is prescribed, all cases of pseudomembraneous candidiasis, oral ulcers, and angular cheilitis are resolved after 3 months (159). However, oral hairy leukoplakia takes longer to respond to the medications but would generally decrease in size after the first month with the eventual disappearance by the end of 5 months (159). It has also been reported that protease inhibitors act directly on inhibiting the production and activity of a major virulence factor of *C. albicans* particularly involved in mucosal (oral and vaginal) candidiasis of HIV-infected individuals (160, 161). Generally, it is believed that reconstitution of the patient’s immune system, characterized by increased in CD4^+^ T cells and decline in viral load, results in the decline of opportunistic infections such as OPC. However, very few patients experience resolution of Kaposi’s sarcoma within the 5-month period post-ART treatment (159).

In another study conducted on 399 HIV-infected ART-naïve patients in India, males were shown to exhibit more oral manifestations over females with a ratio of 1.2:1 (162). Oral candidiasis was found in 77% of the subjects as the most prevalent disease. Erythematous candidiasis was observed to be twofold higher than pseudomembranous candidiasis (162). This is in contrast to another study conducted in a cohort of HIV-positive patients from the South of India (163). In both of these studies, Kaposi’s sarcoma was absent from the list of oral manifestations observed (162, 163). This may be due to homosexuality being a possible risk factor for Kaposi’s sarcoma, while heterosexual practice is the main mode of HIV transmission in Asia (162).

There is a higher probability of acquiring oral manifestations, specifically oral candidiasis, if the CD4 count is less than 200/µL, and therefore, it has been suggested that when a CD4 count is unavailable or unattainable, the presence of oral candidiasis may be a positive predictive value of HIV disease progression (154, 162). It is still unclear as to why oral manifestations resolve so quickly when ART is initiated, and even more uncertain why HPV gets worse. In addition to the increased prevalence of oral warts, HIV-positive individuals are experiencing a higher risk of developing HPV-associated head and neck squamous cell carcinomas and not seeing resolution after commencement of ART (164). HIV-seropositive individuals appear to have a 1.5- to 4-fold increased risk for HPV-associated head and neck cancer (164). It is known that the decline of CD4^+^ T lymphocytes causes the HIV-infected person to become highly susceptible to various infections including the wide range of opportunistic oral manifestations (154, 165). HIV infection may alter local cytokine production, impact oral microbiota, and subsequently virus-induced alterations in the local immune system can impact the mucosal immune response to different oral bacteria. These data suggest OPC susceptibility in HIV patients is predominantly immune based, whereas susceptibility to oral hairy leukoplakia and oral warts may be more associated with factors other than mucosal immune function.

Of interest, HIV-infected individuals are at greater risk of developing Epstein–Barr virus (EBV)-associated malignancy such as non-Hodgkin’s lymphoma (166). However, the potential role of oral innate immunity in pathogenesis of EBV-associated oral lesions in HIV-infected individuals is unknown and merits further investigations.

## HIV and Oral Microbiome

The role of oral microbiota is HIV-infected individuals requires further attention as either the disease (HIV) or the treatment (ART) may impact diversity and composition of the oral microbiome. For instance, it has been shown that elevated viremia in untreated patients is associated with significantly higher proportions of potentially pathogenic *Veillonella, Prevotella, Megasphaera*, and *Campylobacter* species than in healthy controls (109). Interestingly, increased prevalence of these potential pathogens result in the diminished presence of commensal *Streptococcus* and *Veillonella* species (109). However, ART-treated individuals showed lower colonization in their oral cavity by *Neisseria flavescens* compared with healthy controls (109). Another study reported that microbial diversity in the oral cavity of HIV-infected individuals was lower than healthy controls, and this diversity was further reduced following ART treatment (23). By contrast, while the bacterial community composition of oral wash specimens was unchanged in HIV-infected compared with healthy controls, using a deep sequencing approach, a difference in fungal communities was observed (167). A recent study on HIV-infected individuals undergoing ART has reported similar oral microbiomes but significant difference in the composition of the oral cavity microbiota (168). This study found that *Haemophilus parainfluenza*, which has been implicated in opportunistic infections, was associated with the HIV-infected individuals (168). A most recent study utilizing Microarray and pyrosequencing techniques reported a significant difference in the prevalence and distribution of the saliva bacterial communities among HIV-infected individuals before and after initiation of ART (169). Interestingly, this study found that *Actinomyces, Atopobium*, and *Aggregatibacter* genera were significantly different from the baseline after ART (169). These evidence suggest that there is a shift in the oral microbiome and these changes might be associated with HIV infection and/or HIV-treatment and other oral manifestations associated with disease. One of the main limitations of these studies seems to be the limited number of studied subjects. Thus, additional studies on larger cohorts are required to better understand the potential role of HIV infection and/or HIV treatment of oral of microbiome. In addition, how these changes in the oral microbiome can impact on the onset of other opportunistic pathogens in the oral cavity.

## Periodontal Disease and Systemic Inflammation

Inflammation has long been recognized as a powerful contributing factor for systemic conditions, such as CVDs including atherosclerosis, coronary heart disease, and thromboembolic events (107, 170–172). Despite the identification of risk factors such as diet, smoking, obesity, and low physical activity, CVD is still on the rise and attention has turned to other associating factors such as inflammation (108). Recently, a large body of evidence has shown that common oral infections may play an important role in atherosclerosis (170) and an increasingly common chronic inflammatory disease that is present in 10–15% of the world’s population is periodontitis (173).

Periodontitis is a more advanced form of gingivitis where the supporting tissues around the teeth including periodontal ligaments and alveolar bone are breaking down (173). Clinical signs include deepening of the periodontal pocket surrounding the tooth, loss of attachment including the gingival mucosa, which will progressively lead to loosening of the teeth and ultimately tooth loss. The etiology of periodontitis is generally caused by local factors, such as accumulation of dental biofilm from poor oral hygiene practices, but can also be a manifestation of certain systemic diseases. For example, there is a strong correlation between patients who have diabetes mellitus and an increased prevalence of severe periodontitis (173). It is not only more common in these patients but also the progression of periodontitis is more aggressive and rapid (173). In addition, individuals infected with HIV are more susceptible to developing periodontitis because of their compromised immune system. Acute necrotizing periodontitis can also be a manifestation of a newly infected individual with HIV (174). In fact, oral manifestations may be present in up to 50% of HIV-infected individuals, and up to 80% of those who have progressed to AIDS (174, 175).

Looking at periodontitis from a molecular perspective, it appears that the host’s immune system in response to pathogens secretes substantial amounts of pro-inflammatory cytokines, like IL-1β, TNF-α, and IL-6, along with tissue destructive mediators like oxygen intermediates and matrix metalloproteinases (172). The oral mucosal epithelial lining begins to ulcerate and forms an easy port of entry for microorganisms or their by-products into the blood stream (176). Many of these bacteria are Gram-negative, obligate anaerobes and can colonize at distant sites (177). Haraszthy et al. tested 50 samples from patients undergoing carotid endarterectomy to determine if any periodontal bacterial pathogens were present. Forty-four percent of the specimens were positive for at least one periodontal pathogen (177). This suggests that oral microorganisms can enter the blood stream and induce the development and progression of systemic inflammation that may lead to other complications such as CVD.

To further link CVD with periodontitis, specific markers have been examined. In a study of 7,735 cases of fatal and non-fatal coronary heart disease, C-reactive protein (CRP), serum amyloid A protein (SAA), and serum albumin were found to be associating factors with an increased risk of heart disease (107). CRP is produced by the liver and serves as a systemic marker of inflammation. In the general population, a CRP level of less than 1.0 mg/L indicates a low risk for CVD (178). A reading between 1.0 and 2.9 mg/L is an intermediate risk, and a reading greater than 3.0 mg/L is considered high risk (178). A CRP level above 10 mg/L is considered the threshold of significant inflammatory diseases such as cancer, tuberculosis, or an autoimmune disorder (179, 180). In another study of 5,552 participants, it was found that the mean CRP level was approximately one-third higher in subjects that clinically presented with periodontitis than compared to subjects with little or no signs of periodontitis (179). Similarly, significant increase in CRP levels was found in patients with higher levels of clinical attachment loss and bone loss than healthy controls (181). This supports the notion that periodontitis is associated with an increased CRP level that has been correlated with an increased risk of CVD.

In addition to CRP, it has been reported that SAA proteins are also elevated in patients with chronic periodontitis (182). SAA proteins are part of the apolipoproteins group produced mostly by the liver in response to an acute-phase stimulus like an infection (182). A pilot study comparing CRP and SAA levels found that both were elevated in periodontitis patients and SAA, along with CRP, are valuable markers to identify inflammatory conditions in patients (182). Serum albumin is another protein produced by the liver and the presence of inflammation will reduce albumin concentration by decreasing its rate of synthesis (35). The release of inflammatory cytokines, like IL-1, IL-6, and TNF-α during an infection or inflammation, can decrease serum albumin levels (35). In a longitudinal study of 600 subjects, it was found that patients with a serum albumin concentration below 4.0 g/dL had significantly higher rates of periodontitis than patients with a serum albumin concentration above 4.0 g/dL (35).

**Figure 3:**
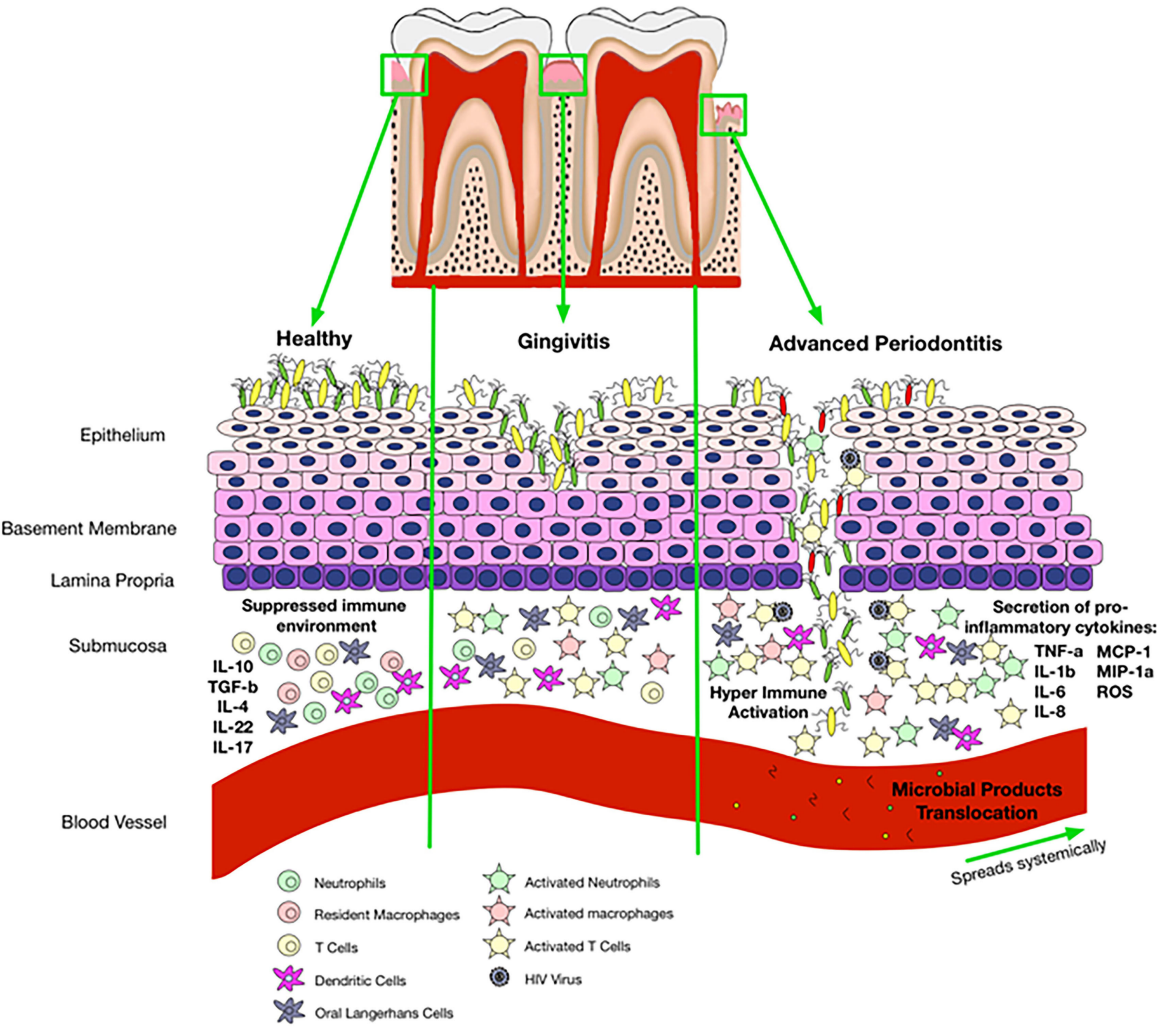
**Proposed mechanisms of oral immune regulation in health versus immune-pathology associated with oral disease and impact of that on HIV acquisition**. In a healthy state, the epithelial cells are able to maintain microbial colonization and there is a suppressed immune environment. Gingivitis disrupts normal immune homeostasis leading to a switch from a suppressed environment to a pro-inflammatory state. Activated immune cells incite pro-inflammatory responses and disturb epithelial tight junctions allowing entry of bacterial products with activation of cells that circulate in the peripheral blood. Once gingivitis has progressed to periodontitis, there is complete breakdown of the epithelium allowing microbial products to enter the blood stream and travel systemically. Disruption of epithelial tight junctions and immune activation also facilitates HIV acquisition in the oral cavity.

As discussed above, patients with periodontitis have higher levels of CRP and SAA and lower levels of serum albumin compared with healthy population. Therefore, these patients might be more prone to the development of CVD. We suggest that the saliva of HIV-infected individuals might have higher levels of CRP and SAA and lower levels of serum albumin but this needs to be further investigated. Thus, these individuals could be at a higher risk of CVD than their non-HIV counterparts because of their compromised immune system. Taken together, we propose that periodontitis and/or compromised oral immune system associated with HIV infection may result in a local inflammatory condition and microbial products translocation that leads to systemic inflammation (Figure 3). In addition, periodontitis and local inflammation in the oral cavity may facilitate HIV acquisition.

## HIV and Immunosenescence

The term “immunosenescence” was first coined by Dr. Roy Walford in 1969 and published in his influential book, “The Immunologic Theory of Aging” (183). His work generated the basis for current ideas about immunological aging. In 1997, Pommier et al. initiated research on immunosenescence in HIV pathogenesis (184). This idea commenced due to the remarkable physiological and immunological similarities between HIV-infected individuals and the HIV-uninfected elderly. It has been observed that during aging, there is a decrease in T cell renewal coinciding with terminally differentiated T cells with shortened telomeres (185). These changes may be due to the downstream effects of immune activation and inflammation causing an overall compromised immune system, eventually leading to immunosenescence. This phenomenon has been observed in HIV-infected individuals at an early age, possibly due to the inflammatory conditions in the GI tract leading to systemic inflammation.

With the introduction of ART, a positive diagnosis of HIV is no longer considered a death sentence. Since HIV patients are now living longer, it has become more obvious that these patients are experiencing accelerated aging (110, 186–189). In other words, these individuals are facing complications that are observed within the elderly population but apparent in a much younger age. They experience non-AIDS related comorbidities, such as cancer, liver and kidney failure, osteoporosis, CVD, as well as neurocognitive decline (189).

One of the features of aging is a depletion in T cell regeneration in addition to terminally differentiated T cells with shortened telomeres (185). It is believed that these changes are due to immune activation and inflammation, leading to a general decline of the immune responses which, eventually translates to immunosenescence (190). A common feature in all of these complications is chronic inflammation which is a hallmark of age-associated comorbidities, as chronic inflammation and immune activation are common features of HIV disease.

The intestinal mucosal tissue is the initial site of HIV replication and acute phase of infection is associated with the vast depletion of CD4^+^ T cells in the gut due to either direct killing by the virus, or due to CD4^+^ T cells apoptosis (9, 191, 192). Unless ART is commenced, the loss of CD4^+^ T cells continues throughout the entire disease course (15). Elimination of CD4^+^ T cells, including Tregs and Th17 cells in the gut results in impairment of the mucosal immune system and the disruption of the GI immune functions (4, 193). Historically, it is observed that HIV-infected individuals experience lymphocyte depletion, malabsorption, and abnormalities of the GI mucosal function (14, 15). Vast changes in the immune system additionally lead to the loss of naïve and memory T cell pool, resulting in a disproportion of T cell phenotypes. This altered state of T cells can impair the homeostatic and regulatory state of the GI tract.

Once an individual is infected with HIV, the immune activation associated with the viral replication results in damage to the epithelial lining of the intestinal mucosa because the GALT contains the majority of CD4^+^ T cells in the body. The virus breaches the intestinal lining of the lamina propria to target and activate CD4^+^ T cells, macrophages, monocytes, and other lymphocytes (194). The activation of the lymphocytes in the gut and the immune response to the virus alters the physical barrier of the epithelial cells (9). The tight junctions of the epithelial cells become inflamed, and the spaces between the epithelial cells become permeable (15). This damage also includes the atrophy or blunting of intestinal villi and crypt hyperplasia (15, 195). Once this occurs, translocation of microbes and their by-products into the lamina propria from the lumen exacerbate inflammation by translocation of microbial products into the periphery (9, 15). The microbial by-products, such as LPS, flagellin, and CpG DNA, induce strong pro-inflammatory responses by activating NODs and TLRs (9). In turn, these directly stimulate peripheral macrophages and DCs to secrete large amounts of pro-inflammatory mediators such as IL-1β, IL-6, and TNF-α (189, 196). Once ART is initiated, these cytokine levels decline, indicating that active HIV replication is indirectly or directly related to this profound inflammatory response (189).

In addition, the gut-associated pathological alterations in HIV-infected individuals results in reduction of IL-17 and IL-22 (14, 15, 197). IL-17 and IL-22 are responsible for maintaining mucosal homeostasis in the GALT but severely compromised upon SIV infection (197). Thus, quantitative loss of Th17 and Th22 CD4^+^ T-cells has been reported to be associated with microbial translocation, systemic immune activation, and disease progression (197). Unfortunately, initiation of ART does not fully restore the functionality and reconstitution of these mucosal Th17, Th22, and Th17/Th22 T-cells (197). In addition to Th22, a subset of gut innate immune cells that express natural killer cell markers referred to as NK-22 provide an innate source of IL-22 and are present in murine mucosa-associated lymphoid tissues and appear in the small intestine lamina propria during bacterial infection (198). Therefore, these innate lymphoid cells (ILCs) are found in mucosa-associated lymphoid tissues and in lamina propria as another source of IL-17 and IL-22 (198). ILCs play fundamental roles in response to infection by producing and secreting cytokines essential for immune regulation, tissue homeostasis, and repair (198). These adaptive lymphoid cells produce several T cell-associated cytokines, but do not express several cell-surface markers commonly seen on other immune cell lineages (199). ILCs do not express a T cell receptor and thus, fail to respond to antigen (199). It appears that ILCs play a role in HIV pathogenesis since ILCs are depleted in the periphery of HIV-infected individuals at early stages of the disease in the absence of ART (200). It does not appear that ILCs are reconstituted if ART is commenced during chronic infection, but depletion of the ILCs can be avoided if ART is initiated during the acute stage of infection (200). However, currently there is no report on oral mucosal ILCs in HIV-infected individuals and whether HIV infection results in depletion of these cells in the oral mucosal as observed in the gut needs to be investigated.

Although depletion of IL-17 and IL-22 is correlated with the extent of microbial translocation, long-term non-progressors and elite controllers are shown to avoid microbial translocation and maintain good levels of CD4^+^ T cells including Th17 cells with low levels of CD4^+^ T cells activation in the gut (197, 201).

## Concluding Remarks

HIV infection could be considered a mucosal disease of the GI tract. The host’s immune response at the mucosal surfaces is a key determinant for HIV acquisition and spread. Thus, compromised mucosal barriers integrity not only facilitates HIV infection but also translocates microbial products into the periphery, resulting in hyper-immune activation. Oral mucosal immunity is alerted during HIV infection, as a result this dysregulated and/or impaired mucosal barrier may predispose the patient to opportunistic infections and systemic inflammation. The studies discussed here evaluate the current knowledge of secretory and cellular components of the mucosal surfaces and their role on HIV pathogenesis. In addition, HIV oral transmission in both infants and adults are discussed and the potential association of periodontal disease with HIV and other opportunist infections are analyzed. Understanding immunological changes at the mucosal compartments, in particular in the oral cavity of HIV-infected individuals in the presence or absence of ART, may assist us to develop novel interventions that may reduce mucosal-associated inflammation. However, further studies are required to determine how the impaired mucosal integrity of oral mucosa due to HIV infection can be prevented or restored. It is likely that such studies of basic mechanisms of oral mucosal barriers and immunological alterations will be essential to provide a foundation for the development of immune therapeutics that can effectively prevent mucosal-associated hyper-immune activation.

## Author Contributions

SE proposed the idea and structured the content. SH wrote the first draft, drew the figures and made the tables. SE edited the drafts and rewrote some sections.

## Conflict of Interest Statement

The authors declare that the research was conducted in the absence of any commercial or financial relationships that could be construed as a potential conflict of interest.
